# Editorial Note: Machine learning–assisted optimization of a terahertz photonic metamaterial absorber for blood cancer detection

**DOI:** 10.1371/journal.pone.0352490

**Published:** 2026-06-26

**Authors:** The PLOS One Editors

The *PLOS One* Editors issue this Editorial Note to inform readers that the works cited in this article [1] as References 11 and 17 were retracted after the article’s [1] publication date. PLOS considers that these references are not crucial in supporting the research or conclusions reported in [1].
